# Hot Isostatic Pressing of Dissimilar 6063Al-SiC/Al and 6063Al-Si/Al Joints for Electronic Packaging: A Comparative Study of Microstructure and Mechanical Properties

**DOI:** 10.3390/ma19214595

**Published:** 2026-10-30

**Authors:** Jie Fang, Yanqiang Liu, Kun Zhang, Chendong Kou, Enze Diao, Jianzhong Fan, Min Yu

**Affiliations:** 1 GRINM Group Corporation Limited, National Engineering & Technology Research Center for Non-Ferrous Metals Composites, Beijing 101407, China; 2 The 29th Research Institute of China Electronics Technology Group Corporation, Chengdu 610036, China; 3 Grinm Metal Composites (Beijing) Technology Co., Ltd., Beijing 101400, China; 4 General Research Institute for Nonferrous Metals, Beijing 100088, China; 5 School of Material Science and Engineering, Southwest Jiaotong University, Chengdu 610063, China

**Keywords:** electronic packaging, functionally graded structure, HIP bonding, structure and mechanical strength

## Abstract

Functionally graded Al-based joints are promising for electronic packaging, yet achieving reliable dissimilar layer bonding remains challenging. Here, we compare 6063Al-SiC/Al and 6063Al-Si/Al joints fabricated by hot isostatic pressing (HIP). Although both achieve defect-free bonding, but the SiC/Al joint exhibits a far higher tensile strength of 253 MPa and an interfacial shear strength of 203 MPa than the Si/Al joint, which reaches only 155 MPa and 48 MPa, respectively. This disparity stems from distinct interface evolution: the SiC/Al joint features an atomically bonded interface with high strain shielding efficiency, confining thermal mismatch strain to a narrow region of approximately 15 µm adjacent to the interface and protecting the 6063Al substrate. In contrast, Si particle fragmentation, imperfect interfaces, and low strain shielding efficiency in the Si/Al joint promote stress concentration and widespread substrate deformation, leading to premature failure with a brittle cleavage mode. These results demonstrate that reinforcement structural stability and strain shielding efficiency of the composite are crucial for the reliability of graded Al joints in electronic packaging.

## 1. Introduction

Owing to the rapid development of electronics and information technology, electronic systems and equipment are increasingly being integrated on a large scale, miniaturized, and made more efficient and reliable [1]. The research and development of innovative electronic packaging materials with excellent performance have been widely studied to meet these requirements, especially the study of thermal management materials. Ideal thermal management materials have a coefficient of thermal expansion (CTE) of 7–9 × 10^−6^ K^−1^, which closely matches that of GaAs or Si, a high thermal conductivity of >100 W/(m·K), and a low density of <3 g·cm^−3^ [2]. Al matrix composites with high-volume fractions of SiC or Si uniquely satisfy all these stringent requirements, simultaneously offering a tailorable CTE that perfectly matches GaAs/Si, making them the most attractive and widely adopted candidates for electrical packaging [3,4,5,6].

The Si/Al composite has attracted considerable attention owing to their excellent combined properties. Its final properties are greatly dependent on the fabrication techniques, including powder metallurgy, spraying deposition, liquid metal infiltration, and spark plasma sintering [7,8,9,10,11]. Recently, a crack-free Al/70Si alloy with a density of 98.8% and compressive strength of 935 MPa was fabricated using selective laser melting [12]. Sr or P modification can further refine the Si particles to the nanometer scale (20–30 nm), significantly enhancing ductility [13,14]. While the SiCp/Al composite with high volume fraction of SiC has become ideal candidates for electronic packaging materials (EPMs) owing to their superior properties, including high thermal conductivity, tailorable coefficient of thermal expansion, and low density in many fields [15,16,17,18,19]. Wiht the help of ultrasonic elutriation to remove ultrafine SiC particles, Sun et al. [19] optimized the hot-pressing process and successfully fabricated 50 vol.% SiCp/6061Al composites with a relative density of 98.6%, a thermal conductivity of 186.3 W/(m·K), a coefficient of thermal expansion as low as 7.9 × 10^−6^/°C (20–100 °C), and a bending strength of 332.4 MPa, demonstrating superior overall performance compared to most previously reported results. Recent years have witnessed a resurgence of interest in functionally graded materials (FGMs), which enable spatial tailoring of the CTE, thermal conductivity, and mechanical properties, offering a potential pathway to overcome the inherent limitations of conventional homogeneous materials. Recent research has primarily focused on the macroscopic tailoring of gradient material properties [20,21,22,23] and interfacial stress issues [24]. For instance, Zhao et al. [23] reported a 60% increase in tensile strength through gradient structures but did not provide a quantitative analysis of stress suppression. Recently, Zhao et al. [24] demonstrated that a graded SiC/Al composite with a tailored CTE gradient significantly reduced the peak thermal stress and maximum warping deformation under rapid thermal cycling, thereby extending the device lifespan.

Owing to the ongoing trend toward miniaturization, lightweighting, unmanned operation, and integration in electronic information systems, electronic packaging faces increasing pressure to further reduce size, weight, and power consumption (collectively referred to as SWaP) [25]. This creates an urgent demand for packaging housing materials that simultaneously combine wide-range thermal matching, excellent brazing and laser welding performance, high thermal conductivity, high specific stiffness, high specific strength, and good machinability. However, high-Si/Al suffers from high brittleness, poor machinability, and large laser welding thermal stresses that narrow the process window, while high-SiC/Al is excessively hard, costly to machine, and prone to poor laser welding hermeticity due to the brittle Al_3_C_4_ phase [26]. Although gradient architectures can redistribute thermal stresses and improve thermo-mechanical compatibility, they do not alter the intrinsic brittleness of SiC/Al composites. The formation of brittle Al_4_C_3_ during fusion welding remains a thermodynamic consequence of the local Al–SiC reaction, which gradient design cannot suppress. Consequently, graded SiC/Al composites still suffer from limited weldability, poor laser welding hermeticity, and machining difficulties similar to those of their homogeneous counterparts.

To address this problem, we have proposed a functionally graded aluminum matrix composites (FGAMCs), a representative class of FGMs, which are fabricated by combining aluminum alloys with materials that share similar lattice types and atomic characteristics but differ in physical properties, yielding a macroscopically integrated composite with continuous micro-interfaces and regionally varying composition and physical properties. By synergizing the advantages of aluminum alloys, high-Si/Al and SiC/Al, even the functionally graded materials, FGAMCs are highly attractive for high-density packaging housings, offering wide-range thermal matching, high thermal conductivity, good fine machinability, and excellent laser welding performance, thereby meeting the demands of high-density packaging. Nevertheless, the successful implementation of such graded structures hinges critically on the reliable joining of dissimilar material layers, which remains a primary challenge. Specifically, when combining Al alloys with Si/Al or SiC/Al composite layers, the differences in thermal expansion and mechanical behavior across the interfaces can lead to severe stress concentration and interfacial failure if the bond quality is inadequate. Therefore, understanding the interfacial bonding characteristics and mechanical performance of the joints is essential for the development of robust FGAMCs suitable for high-density packaging.

In parallel, fabrication techniques play a crucial role in achieving sound joints. A recent review by Sheraf and Gnanaraj [22] highlighted that conventional powder metallurgy (PM) offers a cost-effective and versatile route for fabricating FGAMCs with tailored properties. However, PM faces limitations in full densification, residual stress management, and the creation of complex continuous gradients. These challenges have spurred interest in advanced consolidation techniques such as hot isostatic pressing (HIP). HIP offers superior densification and enables solid-state diffusion bonding, effectively eliminating interfacial porosity and promoting atomic interdiffusion without liquid-phase sintering [27,28]. For example, Luo et al. [28] successfully used HIP to fabricate a SiCp reinforced aluminum matrix composite, achieving full density and excellent mechanical properties. Although these studies demonstrate HIP’s potential for improving interfacial integrity, systematic investigations on the direct joining of the Al alloys to SiC/Al composite are still lacking. This further underscores the necessity of our work, which focuses on the preparation, interfacial characterization, and mechanical assessment of the joints—an essential step toward enabling reliable and high-performance gradient structures for next-generation electronic housing packaging.

## 2. Materials and Characterization

### 2.1. Materials and Experiments

**Figure 1:**
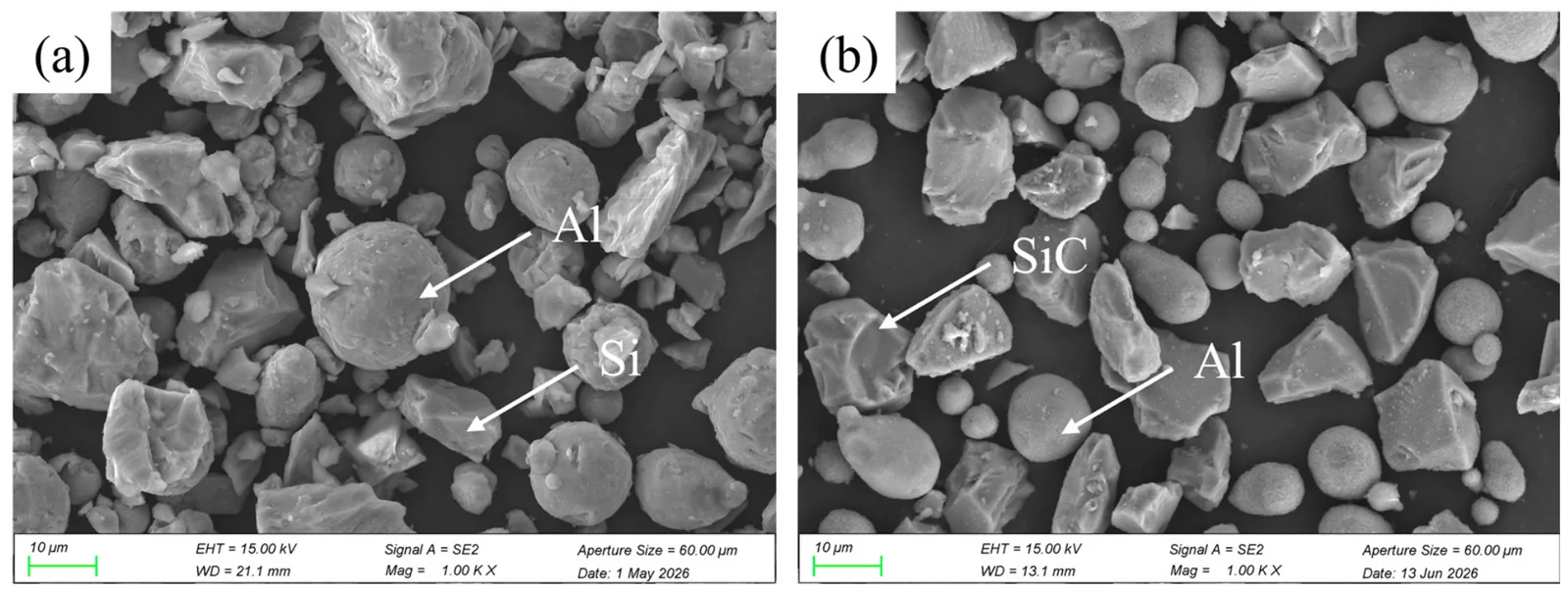
Morphologies of composite powders: (**a**) Al/Si and (**b**) Al/SiC.

In this study, a dissimilar joint between a Si- or SiC-reinforced Al matrix composite and a 6063 Al alloy was investigated. The composite side was prepared by mechanically mixing submicron Si or SiC particles with pure Al powder, with their morphologies shown in Figure 1a,b. The Si and SiC particles had an average size of 15 µm and were supplied by Shandong Qingzhou Micropowder Co., Ltd., Qingzhou, China, at a commercial purity of ≥98.5%. The Al powder had an average size of 10 µm and a purity of ≥99.5%. The volume fractions of Si or SiC were approximately 50 vol.%. Table 1 summarizes the physical properties of materials used for the joint.

**Figure 2:**
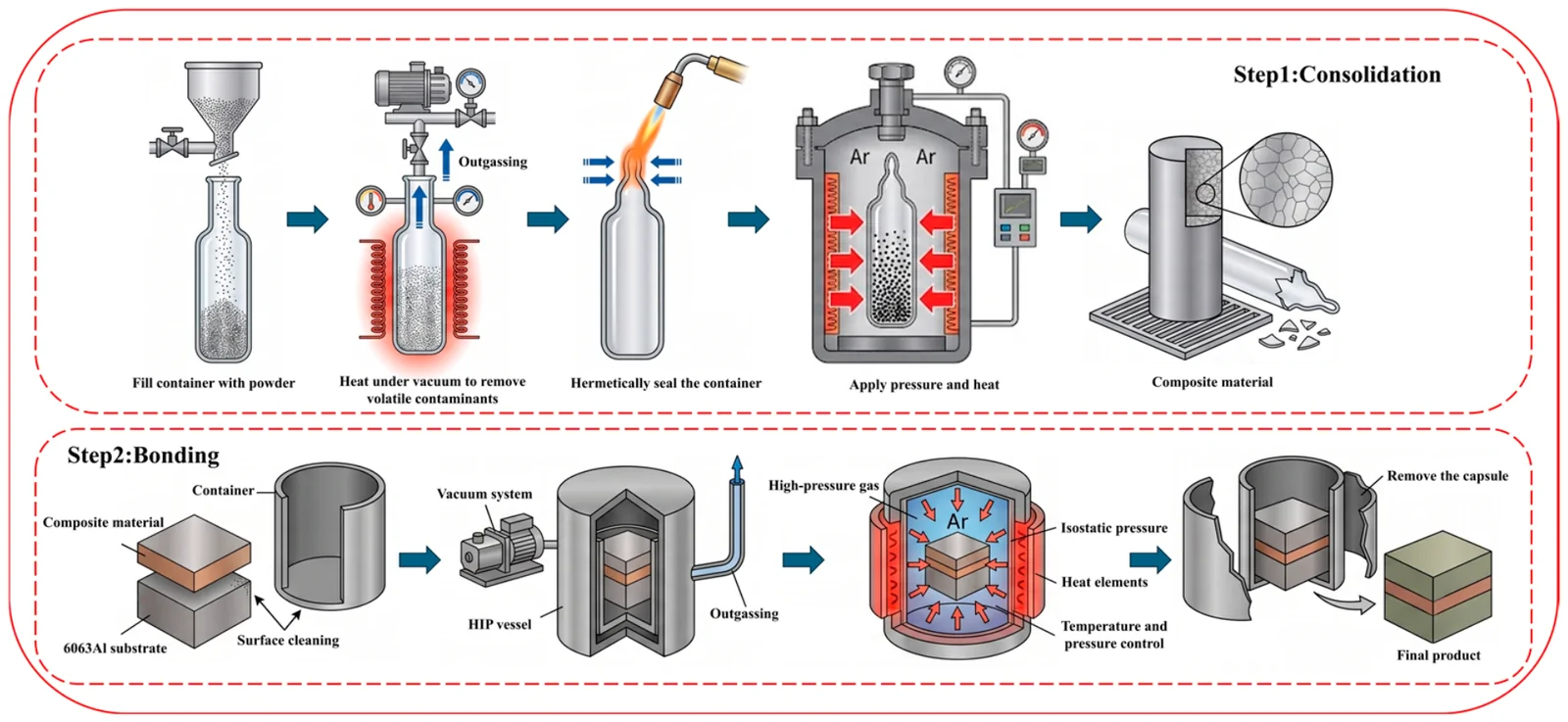
Schematic diagram of HIP manufacturing process. The authors used Gemini (Google) for the purpose of creating schematic illustrations (Figure 2). The authors have reviewed and edited the output and take full responsibility for the content of this publication.

**Figure 3:**
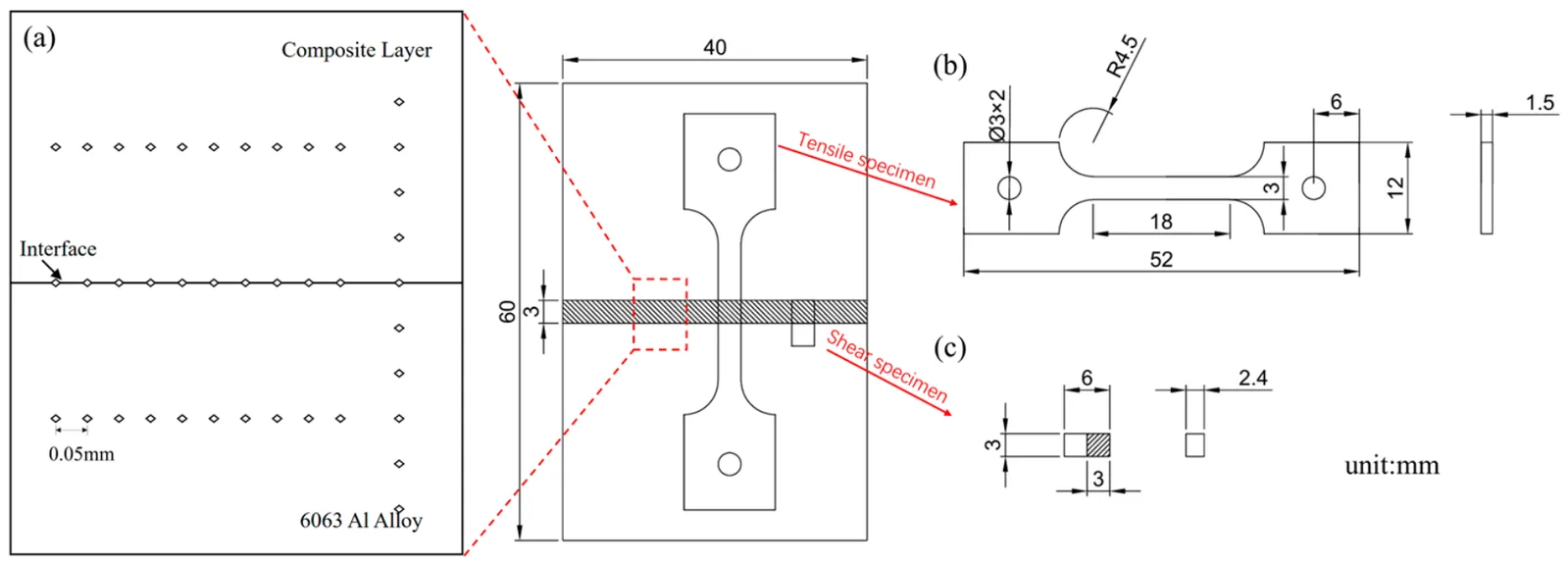
(**a**) Distribution of hardness indentations and dimensions of (**b**) tensile sample and (**c**) interfacial shear strength samples.

The 6063Al-SiC/Al and 6063Al-Si/Al joints were fabricated using a two-step hot isostatic pressing (HIP) process, as shown schematically in Figure 2. The HIP temperature and time were optimized through preliminary experiments to achieve defect-free joints with strengths satisfying electronic packaging requirements. In the first step, the mixed composite powders were consolidated into bulk billets via HIP at 560 °C for 1 h, followed by a second stage at 585–590 °C for 3 h, with a constant isostatic pressure maintained at ≥120 MPa. After the composite layer was prepared, the 6063Al plate and consolidated composite layer were assembled into a sandwich-structured joint. The joint had overall dimensions of 60 mm (length) × 40 mm (width), with the middle composite layer approximately 3 mm thick, as shown in Figure 3. Subsequently, dissimilar joints (6063Al-SiC/Al and 6063Al-Si/Al) were fabricated using a secondary HIP diffusion bonding process. This interfacial joining step was conducted at 530 °C for 1 h, followed by a holding period at 556–560 °C for 3 h, with the isostatic pressure maintained at ≥120 MPa. After the joining process, the joints were solution-treated at 530 °C for 1.5 h, followed by aging at 170 °C for 8 h.

### 2.2. Characterizations

Scanning electron microscopy (SEM) was employed to characterize the macroscopic and microscopic morphologies of the bonded interfaces and the fracture surfaces after tensile testing. The SEM observations were performed at an accelerating voltage of 15 kV, a working distance of 9.7 mm, and an aperture size of 60 µm, using secondary electron (SE2) imaging. The density of the composite layer was measured by the Archimedes method. To map the crystallographic orientation, grain size distribution, and local strain state across the interfaces, electron backscatter diffraction (EBSD) analysis was conducted using an EDAX Hikari Plus system with a step size of 1.2 µm and a working distance of 15 mm. Thin-foil specimens for transmission electron microscopy were prepared using a focused ion beam (FIB) scanning electron microscope, and nanoscale interfacial features and phase boundaries were observed using high-resolution transmission electron microscopy (HRTEM, Thermo Fisher Scientific Talos F200X G2) operated at 200 kV and equipped with a Ceta CMOS camera. The density of the composite layer was measured by the Archimedes method. Microhardness measurements were conducted using a DHV-1000ZTEST microhardness tester under a load of 0.98 N and a dwell time of 15 s. The interfacial shear strength of the joints was assessed via shear testing performed on an electronic universal testing machine (CMT4304, Jiehu Instruments Co., Ltd., Shanghai, China) at a crosshead speed of 3 mm/min. Tensile tests were performed at room temperature using a universal testing machine at a loading rate of 0.1 mm/min. The dimensions of the samples are shown in Figure 3.

## 3. Results and Discussions

### 3.1. Microstructures

**Figure 4:**
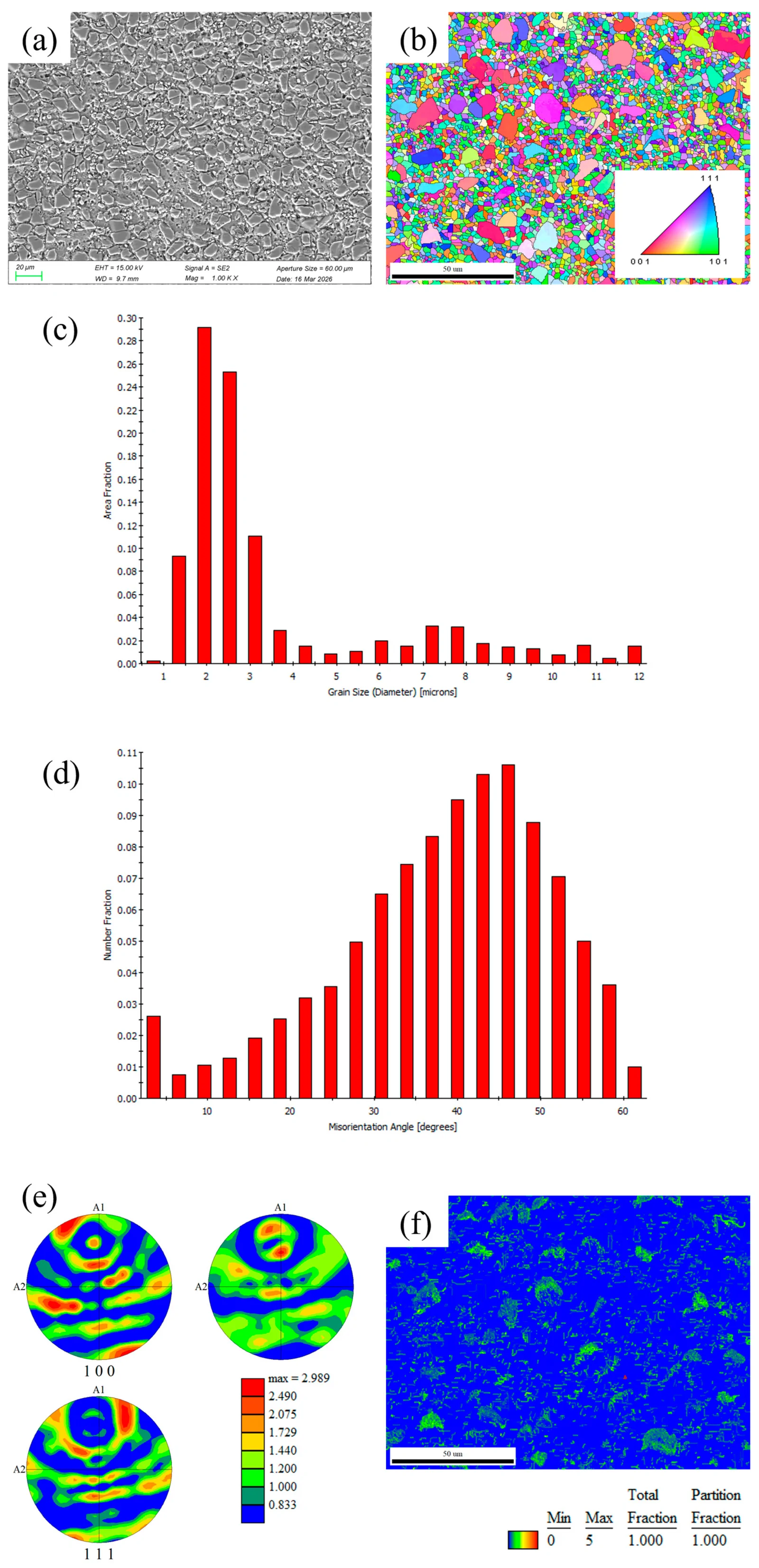
SEM and EBSD results of the SiC/Al composite: (**a**) SEM microstructure, (**b**) IPF, (**c**) grain size, (**d**) misorientation angle, (**e**) Pole figure and (**f**) KAM.

**Figure 5:**
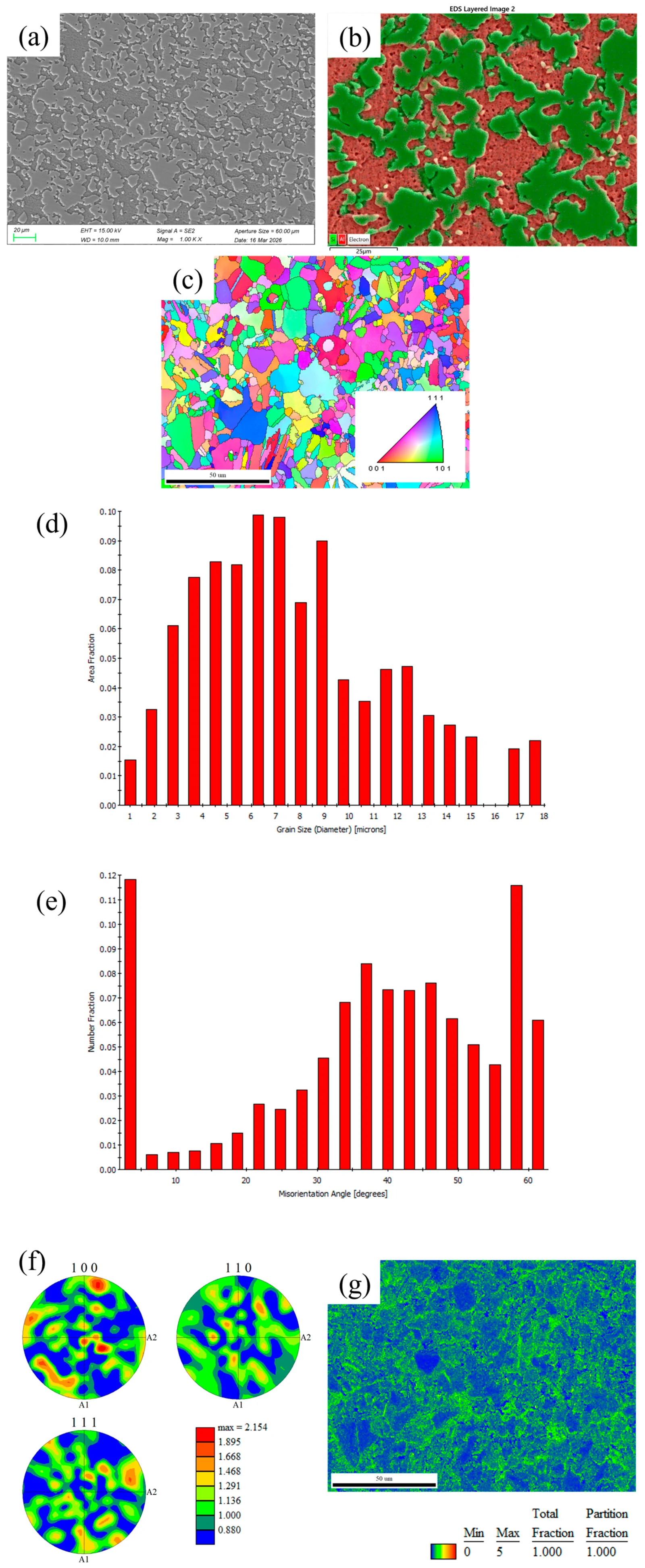
SEM and EBSD results of Al/Si composite: (**a**) SEM microstructure, (**b**) EDS distribution of Si, (**c**) IPF, (**d**) grain size, (**e**) misorientation angle, (**f**) pole figure and (**g**) KAM.

Figure 4 and Figure 5 show the SEM and EBSD results of the SiC/Al and Si/Al composites, respectively. As shown in Figure 4a, the SiC/Al composite exhibits a fully dense microstructure with a relative density of 99.13%, in which angular blocky SiC particles are uniformly distributed and bond tightly to the equiaxed Al matrix. Inverse pole figure (IPF) mapping shown in Figure 4b further exhibits a fully equiaxed grain structure of the Al matrix. Quantitative grain size analysis (Figure 4c) shows a unimodal distribution with a peak area fraction at ~2 µm and a number-grain size of 2.1 µm, indicating a fine and homogeneous Al grain population. The corresponding misorientation angle distribution (Figure 4d) reveals a high fraction of high-grain boundaries (>15°), with a peak number fraction of 10.6% at 46°, and a low proportion of low-grain boundaries (<15°, cumulative fraction < 6%), yielding an average misorientation angle of 38.3°. The pole figure shows a near-random texture with a maximum intensity of only 1.6 (Figure 4e), and kernel average misorientation mapping (Figure 4f) exhibits a low overall misorientation spread (0–5°), indicating a homogeneous strain distribution. These characteristics confirm a fully recrystallized, strain-free Al matrix that is tightly bonded to the uniformly distributed SiC reinforcements. The SEM micrograph of the Si/Al composite (Figure 5) shows a dense microstructure with the relative density of 99.34%, in which the Si particles uniformly disperse in the Al matrix; however, the Si particle exhibits a distinct morphological transformation from the initial angular blocky particles (Figure 1a) into irregular plate-forms, as shown in the EDS map of Figure 5b. The IPF map (Figure 5c) reveals that the composite exhibits a strong preferred orientation, indicating a pronounced crystallographic texture induced by the directional alignment of the plate-Si particle. Quantitative grain size analysis (Figure 5d) shows a broad distribution from 1.0 to 18 µm, reflecting a heterogeneous but predominantly fine matrix. The misorientation angle map (Figure 5e) further indicates that high-angle boundaries (>15°) constitute the majority, with an average misorientation angle of 38.0°, whereas low-angle boundaries (2–5°) account for 11.8%. The KAM map (Figure 5g) therefore displays widespread green regions within the Al matrix, particularly adjacent to the plate-Si particle, indicating severe dislocation pile-ups and strong strain localization. The pole figure (Figure 5f) corroborates these observations by revealing a very sharp single texture component (peak intensity 300× random) on an otherwise random background, a direct consequence of the alignment of plate-Si particle under directional heat flow or extrusion. Comparing to the SiC/Al composite, the morphological transformation of the Si particle causes the higher KAM value in the Si/Al composite (0.63°, compared with 0.15° in the SiC/Al composite shown in Figure 4f), and the sharp edges and high aspect ratio of the plate-like shape could be the primary geometric factors responsible for this significant mismatch.

**Figure 6:**
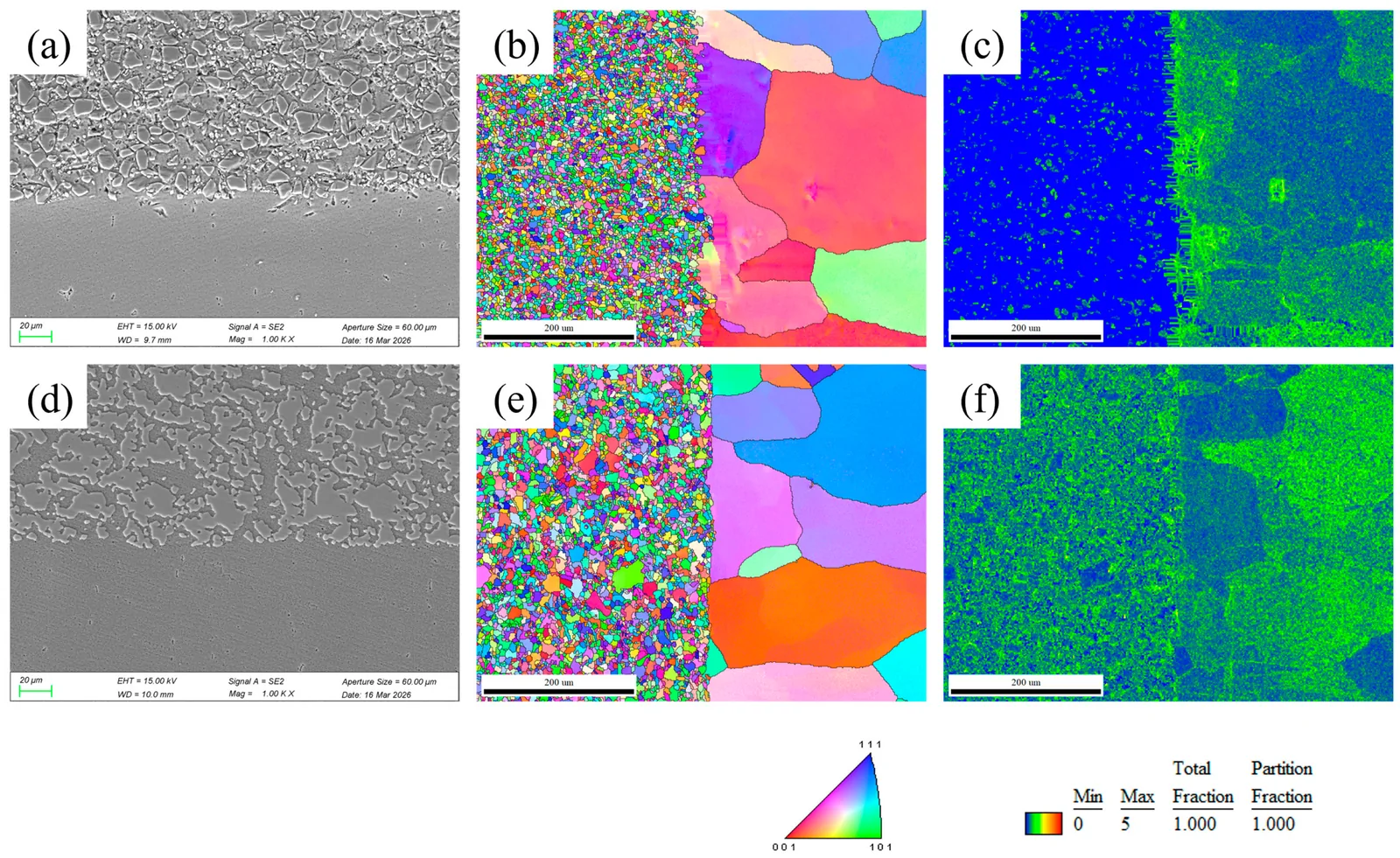
SEM and EBSD results of (**a**–**c**) 6063Al-SiC/Al and (**d**–**f**) 6063Al-Si/Al joints.

SEM and EBSD images of the HIP-bonded 6063Al-SiC/Al and 6063Al-Si/Al joints are shown in Figure 6. Both joints are free from voids and other interfacial defects, demonstrating that interfacial integrity is well preserved. Nevertheless, the strain partitioning between the composite and the 6063Al substrate leads to markedly different kernel average misorientation (KAM) distributions across the interfaces. As for the 6063Al-SiC/Al joint (Figure 6c), a sharp KAM peak is localized precisely at the interface, with the high dislocation density (green) zone extending only ~15 µm into the 6063Al side. Beyond this narrow region, the SiC/Al composite sides remain largely relaxed (blue KAM), indicating that the local lattice distortion and strain localization are confined to a thin interfacial layer. In stark contrast, the 6063Al-Si/Al joint (Figure 6f) displays a diffuse and widespread high-strain distribution. The Si/Al composite side exhibits a pre-existing, uniformly high dislocation density (green KAM), whereas the 6063Al side shows elevated KAM values throughout the entire region.

**Figure 7:**
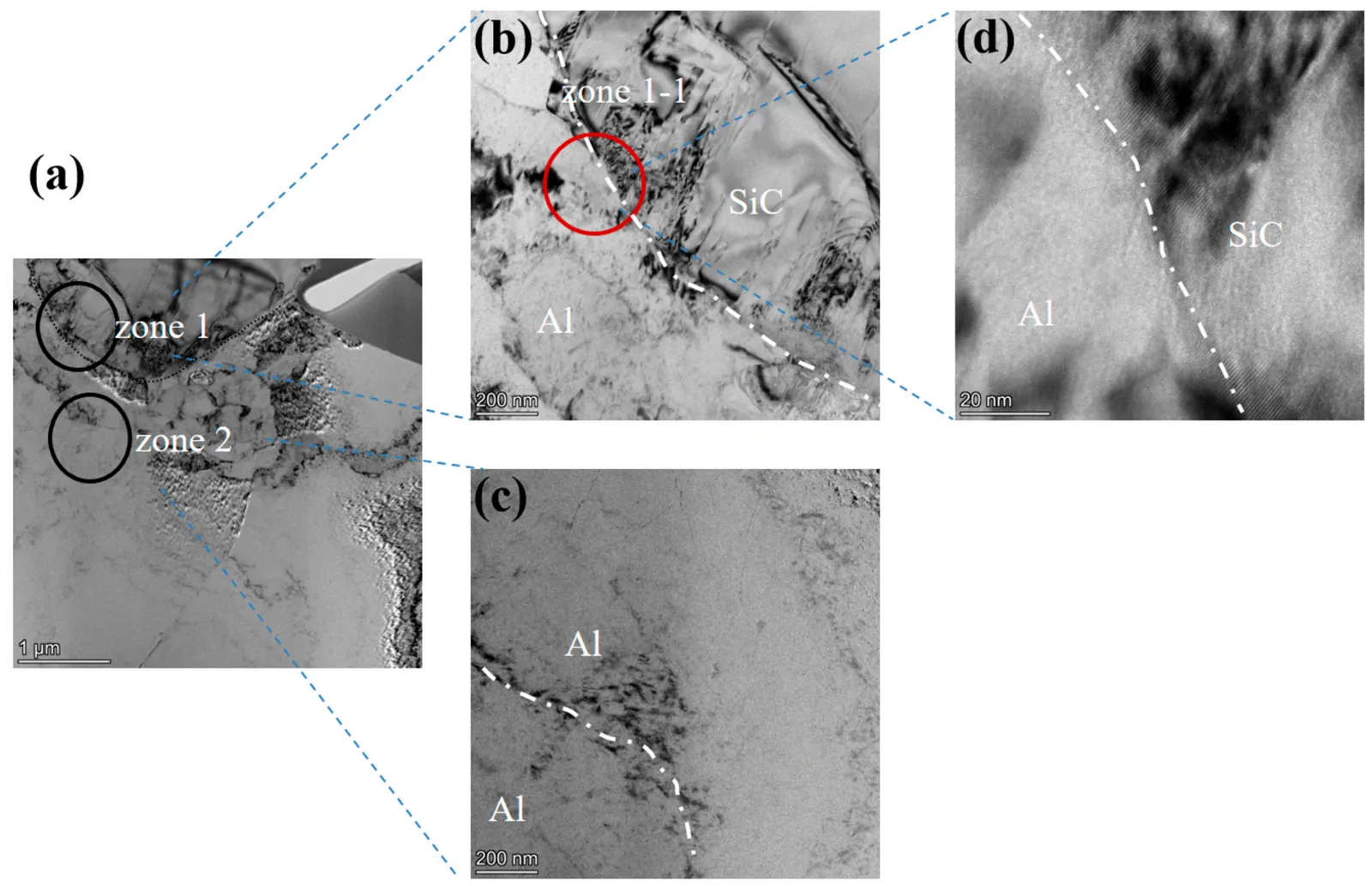
TEM interfacial morphologies of (**a**) Al and SiC, (**b**) enlarged view of zone 1, (**c**) enlarged view of zone 2, and (**d**) enlarged view of zone 1-1.

**Figure 8:**
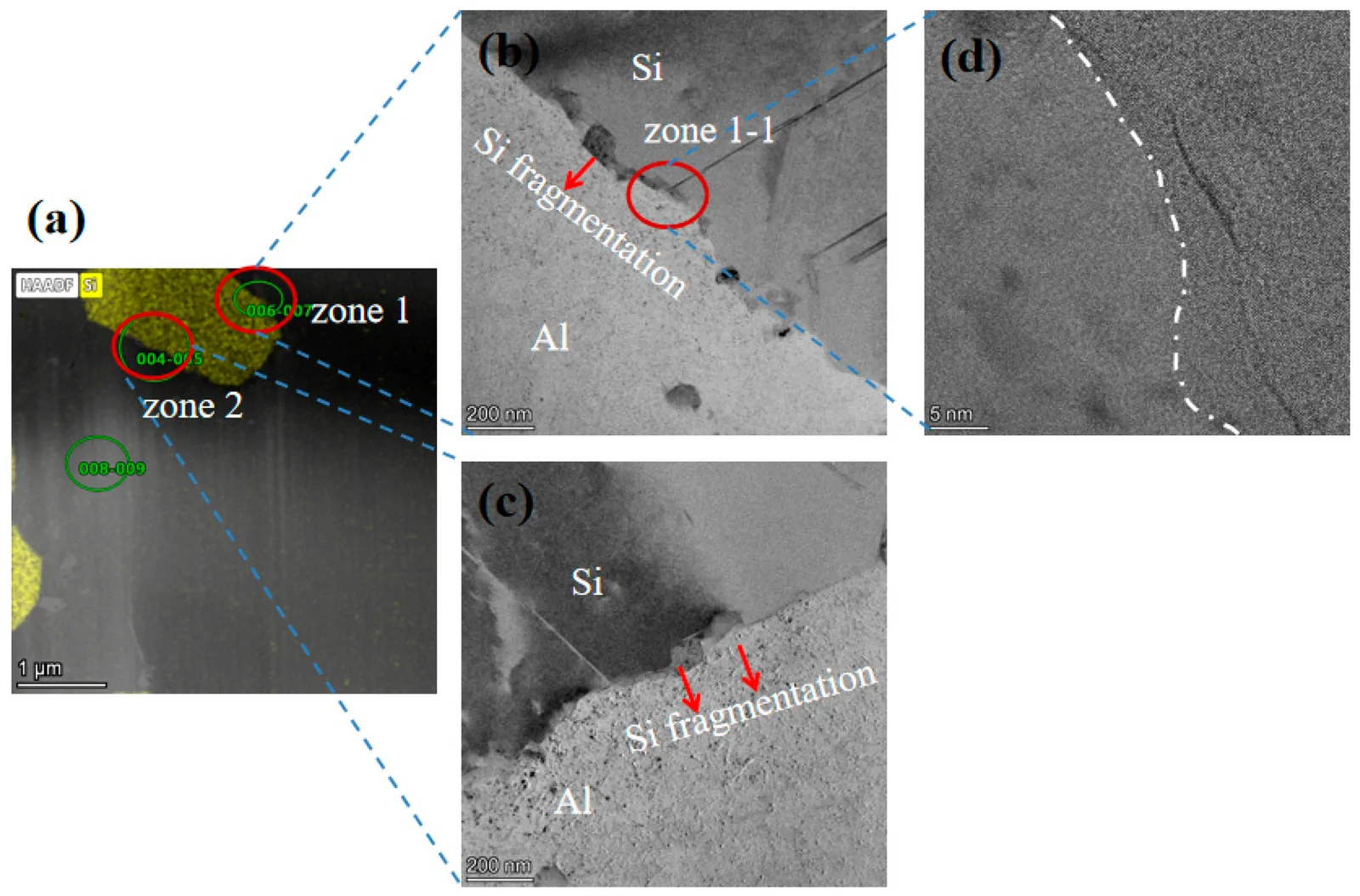
TEM interfacial morphologies of (**a**) Al and Si, (**b**) enlarged view of zone 1, (**c**) enlarged view of zone 2, and (**d**) enlarged view of zone 1–1.

To clearly present the interfaces between the reinforcements of Si and SiC with the Al matrix, TEM characterizations of the SiC/Al and Si/Al composites are shown in Figure 7 and Figure 8, respectively. The SiC/Al interface exhibits a fully dense and continuous morphology, characterized by the absence of voids or unbonded regions (Figure 7a), confirming the high-quality consolidation during the two-step HIP cycle. Careful observation at high magnification, as shown in Figure 7b, reveals that the Al matrix adjacent to the SiC particles contain a high density of parallel dislocation lines and tangles, indicating effective dissipation of the thermal mismatch stress via dislocation generation and local plastic deformation in the Al side. The HRTEM image (Figure 7c) further shows that the lattice fringes of Al and SiC are in direct atomic contact, achieving a strong metallurgical bonding. These features enable efficient stress transfer from the matrix to the reinforcements. In contrast, the TEM observation of the Si/Al interface (Figure 8) shows the localized fragmentation of the Si particles, predominantly confined to the regions adjacent to the interface of the Al and Si particles (Figure 8b,c), along with the penetration of the Al matrix between the fragments. Furthermore, its HRTEM image (Figure 8d) shows the localized lattice distortion in the Si crystal near the crack tips and fragment edges, confirming that the crushing process induced not only brittle fracture but also limited plastic deformation or lattice disordering in the Si particle.

**Figure 9:**
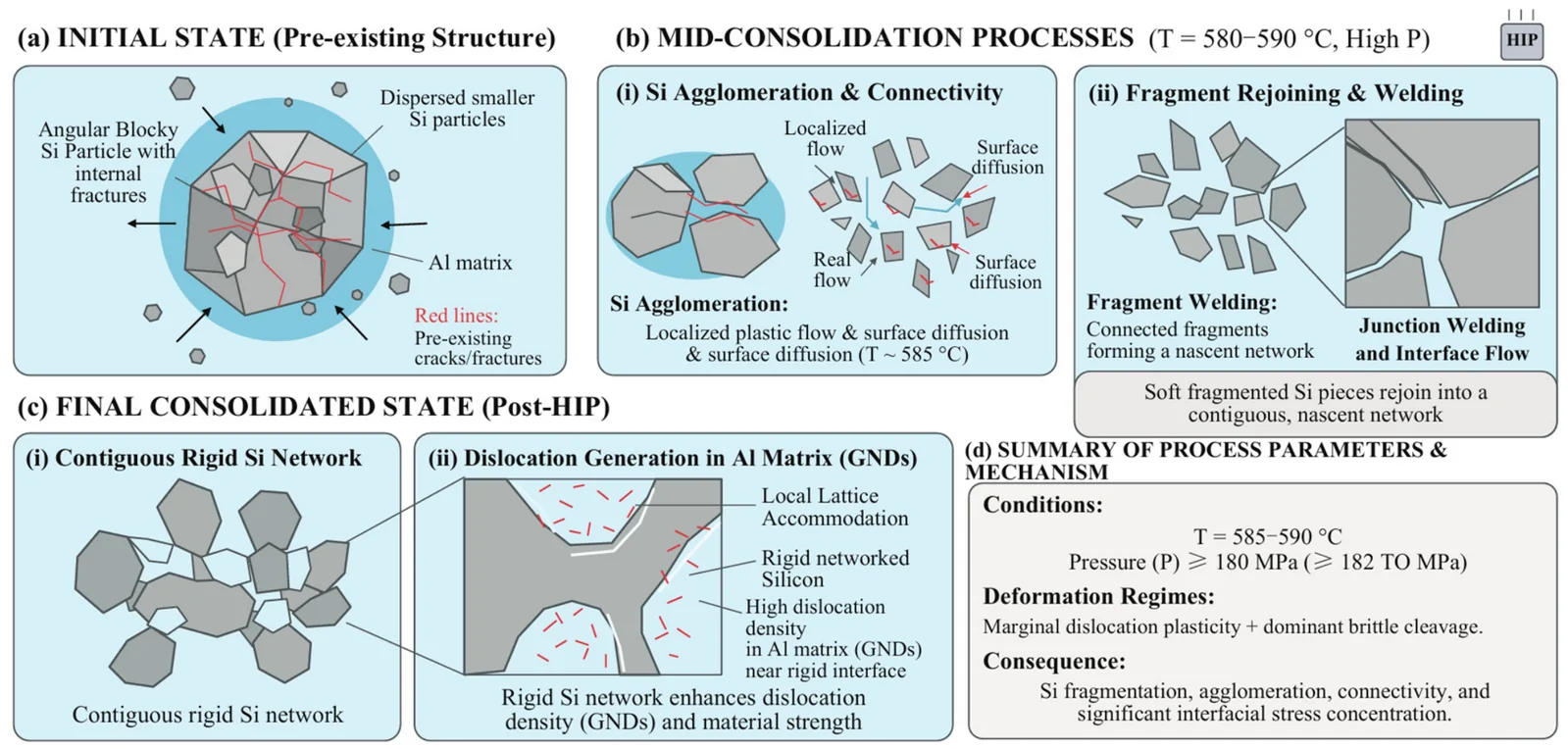
Schematic illustration of the Si particles morphology change during HIP. The authors used Gemini (Google) for the purpose of creating schematic illustrations (Figure 9). The authors have reviewed and edited the output and take full responsibility for the content of this publication.

SEM, EBSD, and TEM observations consistently reveal that the initially angular blocky Si particles in the Si/Al composite underwent severe fragmentation during HIP. The Si particles were transformed into irregular plate shapes (SEM, Figure 5), with small fragments dispersed along the Si/Al interface and localized lattice distortion within the Si crystals (TEM, Figure 8). The EBSD map shows a high density of dislocations concentrated at the Si/Al layer, indicating that the Al matrix accommodates the strain arising from the behavior of the Si particles. To clearly present the behavior of Si in HIP, a schematic illustration of the morphological evolution of Si particles is shown in Figure 9. First, the deformation of the Si particles is attributed to the unique thermomechanical conditions of the HIP cycle. Although Si is conventionally regarded as brittle at room temperature, it undergoes a ductile-brittle transition at elevated temperatures with bulk Si begining at approximatedly 540 °C [29]. In the present HIP process, the second isothermal holding at 585–590 °C under a hydrostatic pressure of ≥120 MPa placed Si in a regime where dislocation plasticity becomes marginally active, yet the material remained predominantly brittle. The high isostatic pressure was effectively transmitted to the dispersed Si particles through the soft, creep-aluminum matrix. Consequently, the Si particles experienced severe compressive deformation, which triggered crack initiation along the preferred cleavage planes, notably the {111} family, leading to fragmentation into irregular plate fragments, as shown in Figure 9a. In addition to fragmentation, elevated temperature and pressure promoted inter-contact of the Si particles. Figure 9b shows that two scenarios could occur: (i) originally separate Si particles were pressed directly together without fracturing, forming larger Si agglomerates; (ii) fragmented Si pieces were compacted and re-united under pressure, creating a contiguous Si network via surface diffusion or limited plastic flow. These interconnected Si structures further exacerbated the local stress concentration because the rigid Si network transfers the load more efficiently to the Al matrix over a longer range as shown in Figure 9c. This mechanism (Figure 9d) is consistent with the deformation and fragmentation behaviors observed in other Si/Al composites processed by HIP [30]. The resulting fragmented yet partially connected Si morphology, together with the imperfect Si/Al interface, explains the extensive strain localization and elevated KAM values measured in the Si/Al composite.

### 3.2. Hardness of the Joints

**Figure 10:**
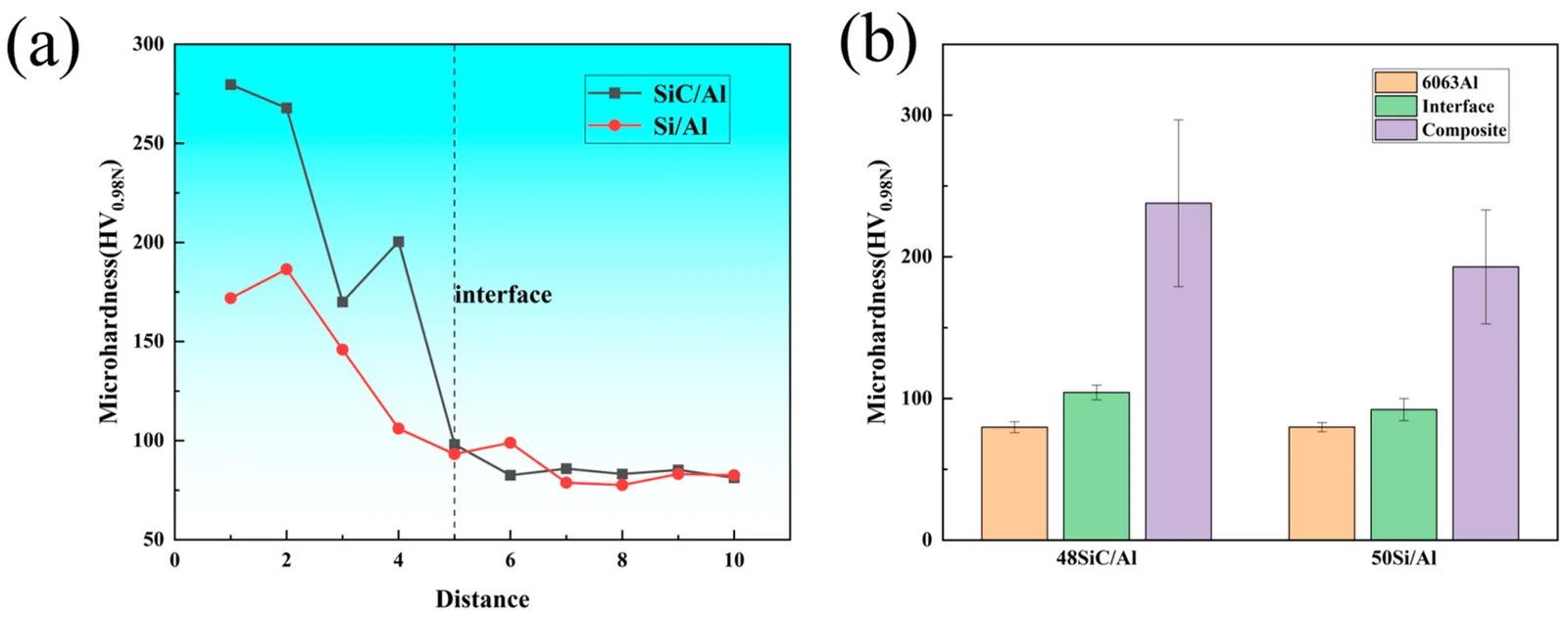
(**a**) Microhardness evolution with distance and (**b**) comparison of average hardness.

Figure 10 shows the microhardness of the joints. Along a longitudinal path from the composite layer toward the 6063Al, the microhardness decreases smoothly from over 200~270 HV to approximately 80 HV (average hardness of 6063Al), as shown in Figure 10a. From the average values shown in Figure 10b, the SiC/Al composite layer exhibits an average hardness of approximately 235 ± 50 HV, whereas the Si/Al composite layer exhibits a lower average hardness of 195 ± 40 HV. In the interfacial transition zone, the average microhardness of the SiC joint is approximately 105 HV, which is higher than that of the Si joint (≈95 HV). The higher interfacial hardness of the SiC joint is attributed to three factors: the intrinsically higher hardness of SiC particles shown in Table 1; the absence of Si particle fragmentation and the beneficial strain hardening caused by residual strain concentration at the SiC/Al interface.

### 3.3. Shear and Tensile Strength of the Joints

**Figure 11:**
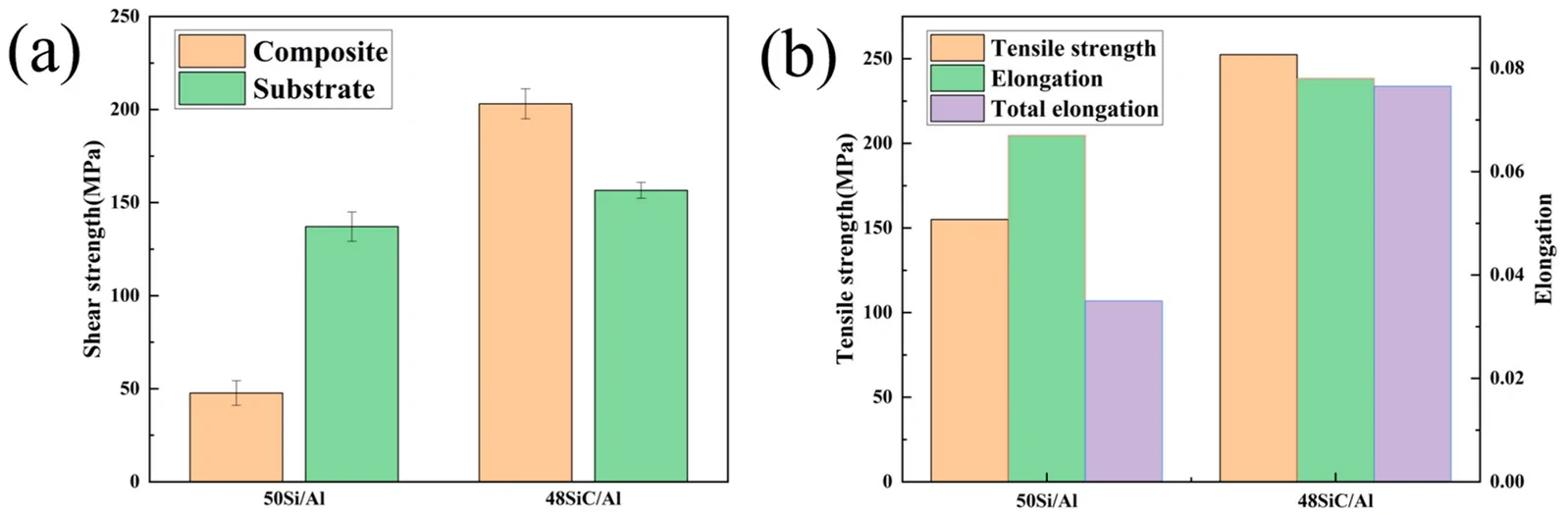
(**a**) Shear strength and (**b**) tensile strength of the joints.

**Figure 12:**
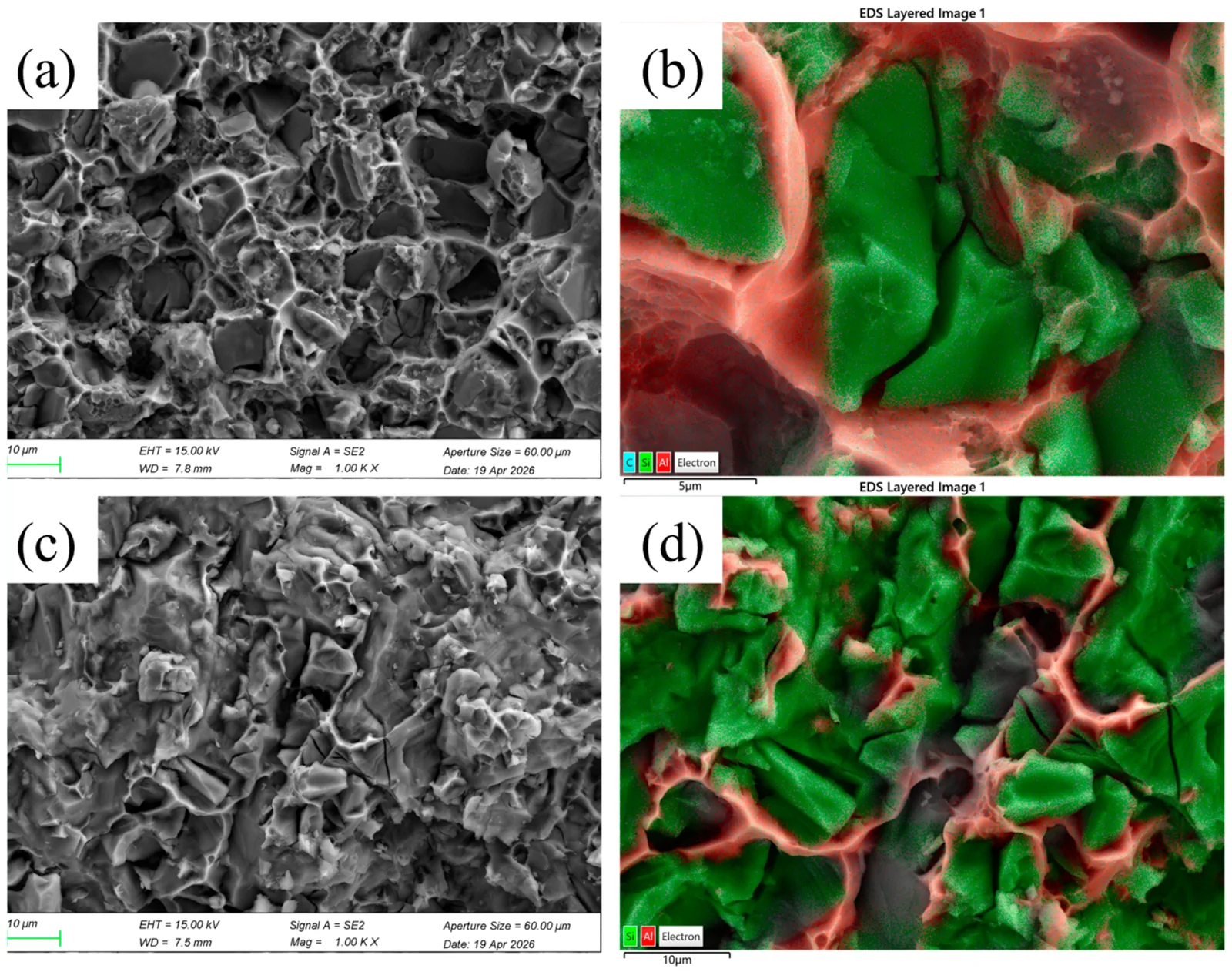
Tensile fracture morphologies and EDS analysis of (**a**,**b**) the SiC/Al joint and (**c**,**d**) Si/Al joint.

Figure 11 shows the shear and tensile strengths of the joints. The shear test results (Figure 11a) reveals a marked difference in the interfacial bonding quality between the two joints. The Al6063-SiC/Al joint exhibits an average interfacial shear strength of 203 ± 8 MPa, reaching ~130% of the 6063Al base metal strength (157 ± 3 MPa). In contrast, the Al6063-Si/Al joint exhibits a significantly lower value of only 48 ± 5 MPa, corresponding to ~35% of the base metal strength. The macroscopic load capacities of the two functionally graded joints were also quantitatively evaluated via room-temperature uniaxial tensile testing. The SiC/Al joint exhibits a superior tensile performance (Figure 1b), with an average ultimate tensile strength of 253 ± 4 MPa, an elongation after fracture of 7.8% (for the dissimilar materials), and a total elongation of 7.7%. In contrast, the Si/Al joint exhibits markedly lower values of 155 ± 3 MPa, 6.7%, and 3.5%, respectively. Fracture tensile surface observations (Figure 12) reveals distinctly different failure mechanisms. The fracture surface of the SiC/Al joint exhibits extensive fine dimples within the Al matrix, indicative of pronounced plastic deformation and substantial energy dissipation. Fragmented SiC particles, rather than clean particle pull-outs confirms effective load transfer from the matrix to the reinforcement. This is supported by the TEM observations of a clean, atomically bonded SiC/Al interface and EBSD evidence of dislocations accumulating in the adjacent Al matrix, both of which enable efficient strain accommodation and stress transfer. Notably, the corresponding photographs show small amounts of the matrix remained on the SiC_p_ surface. This phenomenon was also observed by Wu et al. [31], who suggested that the failure was caused by debonding of the interface.

In contrast, the Si/Al joint (Figure 12c,d) displays a flat, cleavage-domination morphology with large exposed Si facets (identified by EDS mapping) and negligible plastic flow in the Al matrix. This brittle failure mode is directly correlated with the microstructural features revealed by TEM: fragmented Si particles, lattice distortion, and imperfect Si/Al interfaces, which act as preferential crack initiation sites. The severe strain localization captured by EBSD-KAM further confirms that the Al matrix near the Si/Al interface cannot effectively relax the local stress concentration. Consequently, cracks propagate rapidly along the interface with minimal energy absorption, leading to the drastically reduced tensile strength and ductility of the Al6063-Si/Al joint.

## 4. Discussion

The preceding mechanical tests revealed distinct differences between the two joints, suggesting that strain localization at the interface may play a critical role in governing the overall performance. To qualitatively interpret these differences in terms of strain distribution, we adopt the concept of strain partitioning to describe the observed KAM variations and differences in tensile strength. The concept of strain partitioning has been defined at different length scales in previous studies. At the microstructural scale, Oh et al. [32] defined strain partitioning as the phenomenon in which “plastic strain is distributed differently among the present microstructural constituents” (phase-strain partitioning). At the intermediate layer scale, Singh and Jonnalagadda [33] extended this concept to multi-steel sheets, describing the non-distribution of plastic strain across alternating austenitic and martensitic layers (layer-strain partitioning). In the present study, we further extended this concept to the macroscopic scale and defined a macro strain partitioning coefficient *η* to quantify the difference in plastic strain accumulation between the composite side and the adjacent 6063Al substrate across the bonded interface. This coefficient captures the efficiency of the strain shielding provided by the composite layer and serves as a direct metric for comparing the interfacial strain localization behaviors observed in different dissimilar joints.

(1) $$\eta \propto \frac{\sigma_{y,\mathrm{comp}}}{\Delta \alpha \cdot \Delta T\cdot K_{t}}$$

where *σ*_y,comp_ is the yield strength of the composite, Δ*α*⋅Δ*T* is the thermal mismatch strain, and *K_t_* is the stress concentration factor associated with the reinforcement morphology.

**Figure 13:**
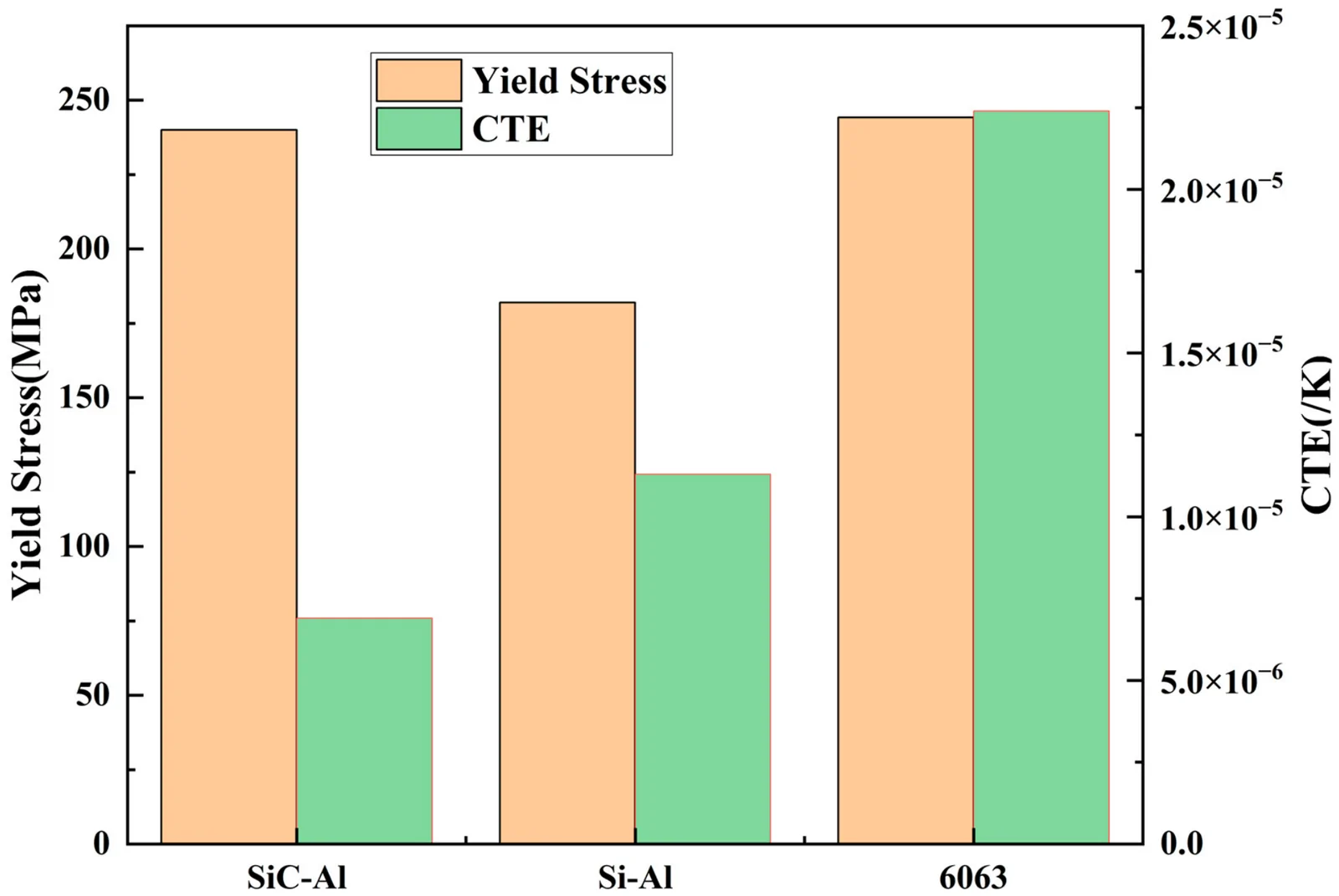
Comparison of yield stress and CTE between the composites.

A qualitatively comparison of the two composites was performed using the measured physical properties shown in Figure 13, which reveals a higher *η* for the SiC/Al composite than that for the Si/Al composite. Specifically, the SiC/Al composite possessed a higher yield strength (~240 MPa vs. ~182 MPa for Si/Al), but a larger CTE mismatch with 6063Al (Δ*α* ≈ 16 × 10^−6^ K^−1^ vs. ~12 × 10^−6^ K^−1^ for Si/Al). These two effects were largely counterbalanced, yielding a comparable strength-to-CTE-mismatch ratio for the two composites. In addition, the SiC/Al composite possessed a lower stress concentration factor owing to its equiaxed blocky SiC particles, in contrast to the sharp plate-like morphology in the Si/Al composite. Consequently, the SiC/Al composite achieves a high strain shielding efficiency, absorbing most of the thermal mismatch strain locally near the interface and transmitting only a minor stress to the 6063Al side, hence the narrow (~15 µm) high zone. In contrast, the Si/Al composite exhibits a low *η* because its larger Δ*α* generates a higher thermal stress, its lower *σ*_y,comp_ limits local strain accommodation, and its anisotropic plate-Si particle act as potent stress concentrators (high *K_t_*). Consequently, the Si/Al composite could not shield the 6063Al substrate; instead, it forced the 6063Al side to plastically deform over a large distance (>200 µm), producing the observed diffuse high-strain KAM distribution.

The macroscopic load capacities of the two functionally graded joints were quantitatively evaluated via room-temperature uniaxial tensile testing (Figure 11b). These differences in tensile behavior could be directly rationalized by the *η* values of the two joints. For the high-*η* SiC/Al composite, the effective strain shielding ensures that the 6063Al substrate is protected from excessive plastic deformation, allowing the composite layer to bear the majority of the tensile load up to the fracture strength of the SiC reinforcements. This load-transfer efficiency enables the joint to achieve a high ultimate tensile strength while maintaining appreciable ductility. In sharp contrast, the low-*η* Si/Al composite fails to provide adequate strain shielding; instead, the inability to accommodate thermal mismatch strain locally forces the adjacent 6063Al substrate to deform plastically, which not only reduces the effective load-bearing cross-section of the joint but also promotes early strain localization and premature failure. Consequently, the Si/Al joint exhibits both lower tensile strength and inferior ductility. Finally, the fracture surface observations (Figure 12) further corroborate this *η*-based interpretation. The high-*η* SiC/Al joint displays a ductile fracture morphology characterized by extensive fine dimples with fragmented SiC particles, indicating that the interface remains intact while the Al matrix undergoes substantial plastic tearing—a signature of effective load transfer and strain shielding. The tortuous crack paths arising from crack deflection at the three-dimensional SiC/Al interfaces further retard crack propagation and enhance energy dissipation, consistent with its superior tensile performance. In contrast, the low-*η* Si/Al joint fails by a brittle cleavage mechanism, exhibiting large, exposed Si facets and negligible matrix plasticity. The intense strain localization at the imperfect Si/Al interface facilitates rapid crack propagation with minimal energy absorption, confirming that the insufficient strain shielding leads to catastrophic interfacial failure. Thus, *η* not only rationalizes the differences in KAM distribution but also serves as a unified metric linking microscale strain partitioning to macroscale tensile performance and fracture behavior, underscoring that the strain-shielding efficiency of the composite layer could be one of the key factors determining the strength and failure mode of the joints.

## 5. Conclusions

In this study, two functionally graded joints, 6063Al–SiC/Al and 6063Al–Si/Al, were successfully fabricated by hot isostatic pressing (HIP) and systematically characterized. The main conclusions are as follows:

(1) Both functionally graded joints were successfully fabricated by HIP. In the 6063Al–SiC/Al joint, the SiC particles retained their equiaxed blocky morphology and formed a clean, atomically bonded interface with the Al matrix. In contrast, the initially angular blocky Si particles in the 6063Al–Si/Al joint underwent severe fragmentation during HIP, transforming into irregular plate-like fragments dispersed along the interface, accompanied by localized lattice distortion within the Si crystals.

(2) The SiC/Al joint confined strain localization to a narrow region (~15 µm) adjacent to the interface, whereas the Si/Al joint exhibited extensive strain localization in the 6063Al substrate over a broad region exceeding 200 µm. This contrast reveals that the reinforcement phase controls the strain shielding efficiency of the composite layer and thus governs the deformation compatibility of the joint.

(3) The SiC/Al joint achieved an ultimate tensile strength of 253 MPa and an interfacial shear strength of 203 MPa, with a ductile fracture morphology characterized by fine dimples. In contrast, the Si/Al joint exhibited only 155 MPa and 48 MPa, respectively, with a brittle cleavage failure mode. The results demonstrate that the structural stability of the reinforcement governs both the interfacial bonding quality and the strain shielding efficiency of the composite, thereby determining the mechanical reliability of graded Al joints.

## Figures and Tables

**Table 1:** Physical properties of materials used for the joint [15].

Parameters	Al	SiC	Si	Al6063
Density (g/cm^3^)	2.7	3.2	2.3	2.7
Melting point (°C)	660	2700	1414	660
Hardness (HV)	33	~2500	~1150	80
CTE × 10^−6^/K	23.2	3.7	4.1	23
Thermal Conductivity W/(m·K)	237	290	150	200

## Data Availability

The original contributions presented in this study are included in the article. Further inquiries can be directed to the corresponding authors.
